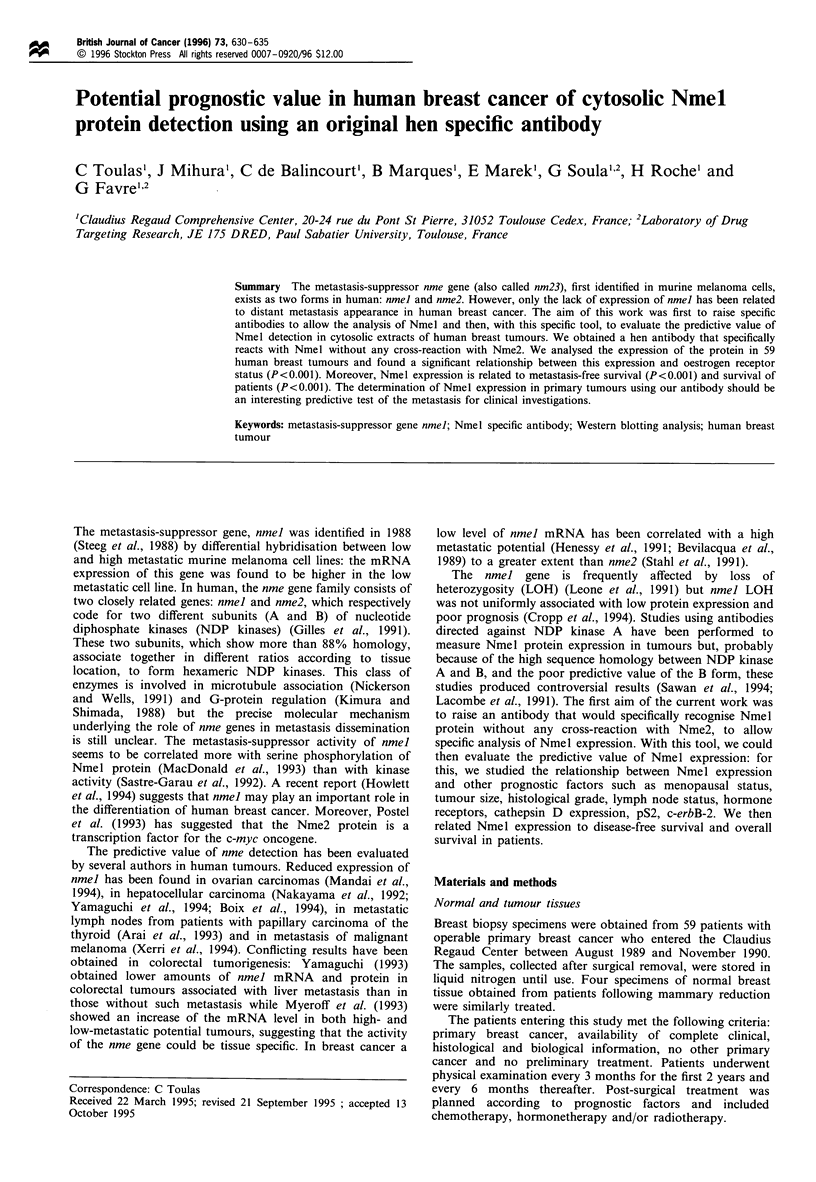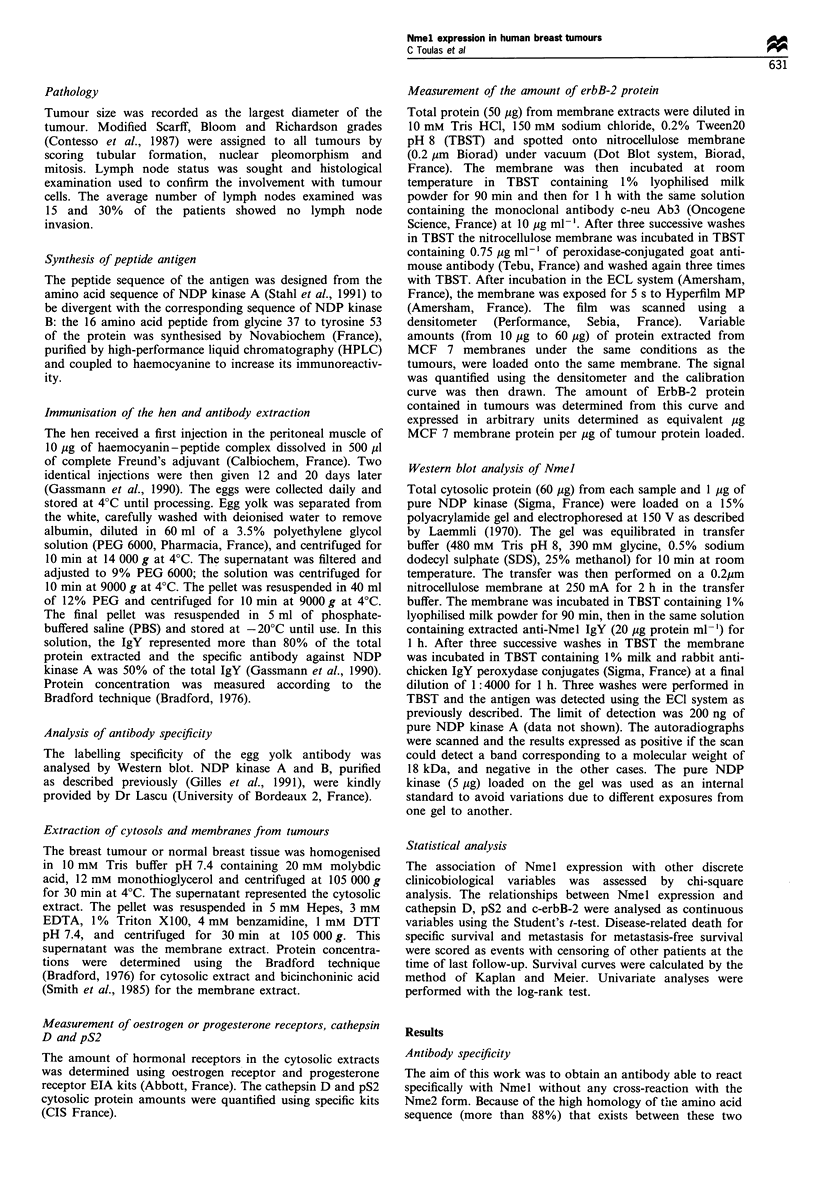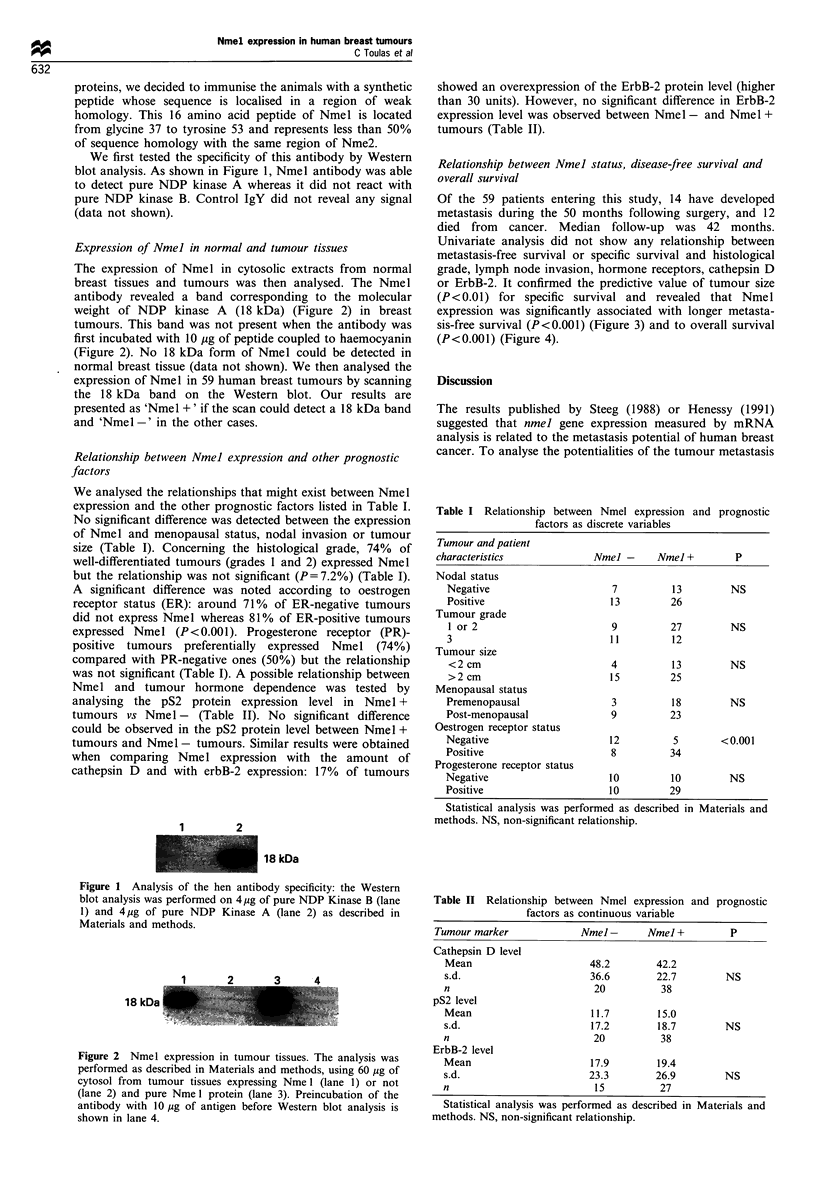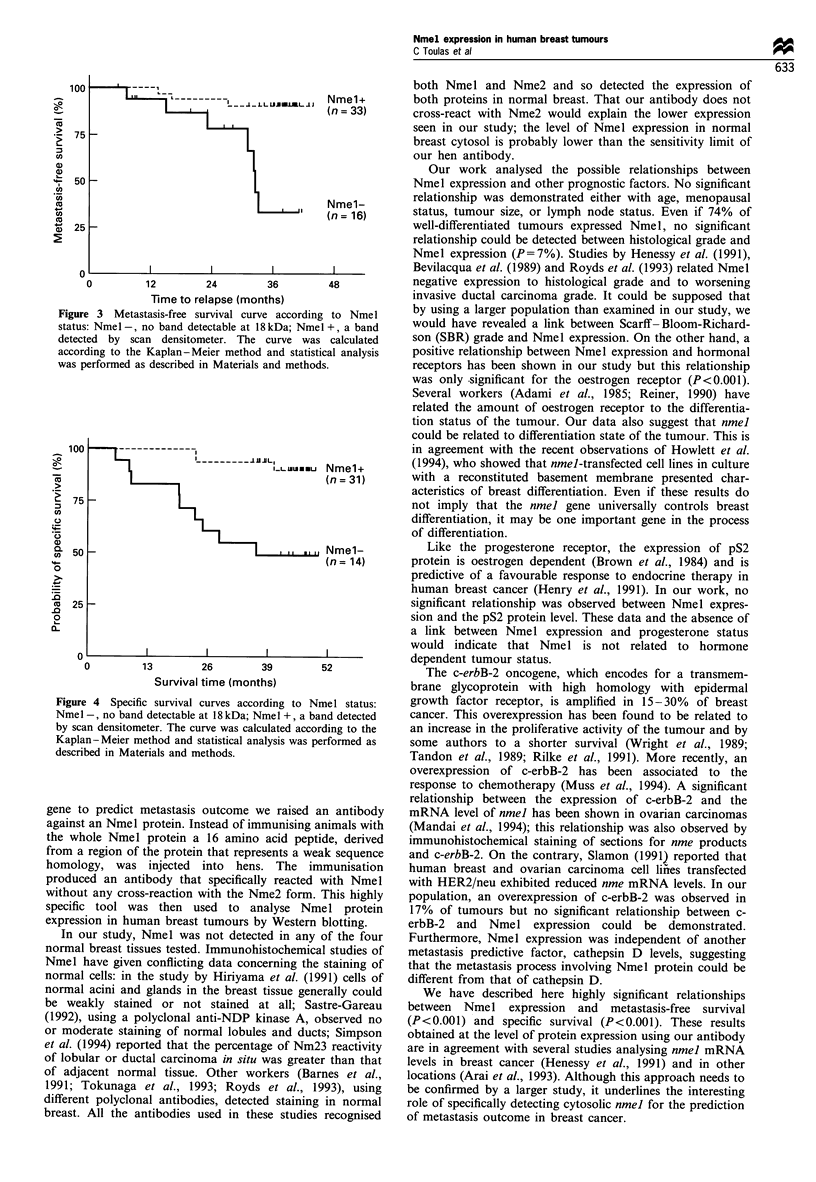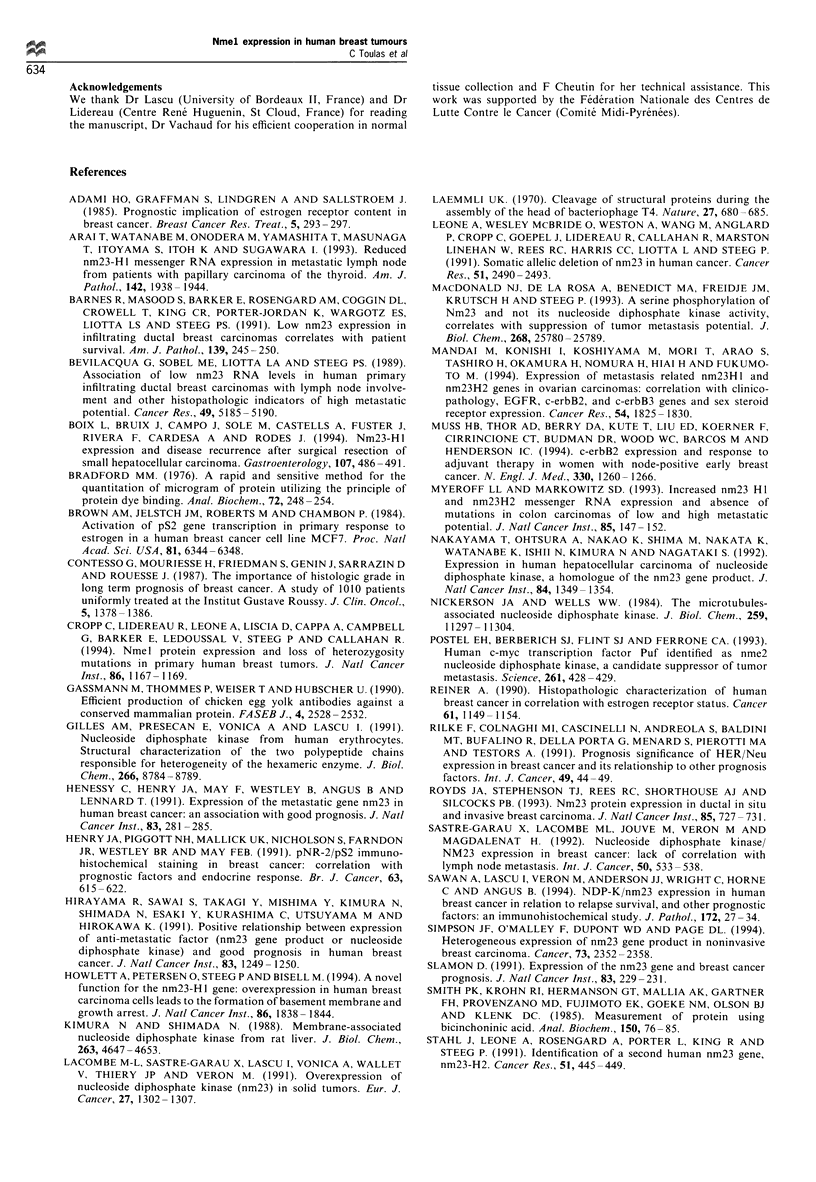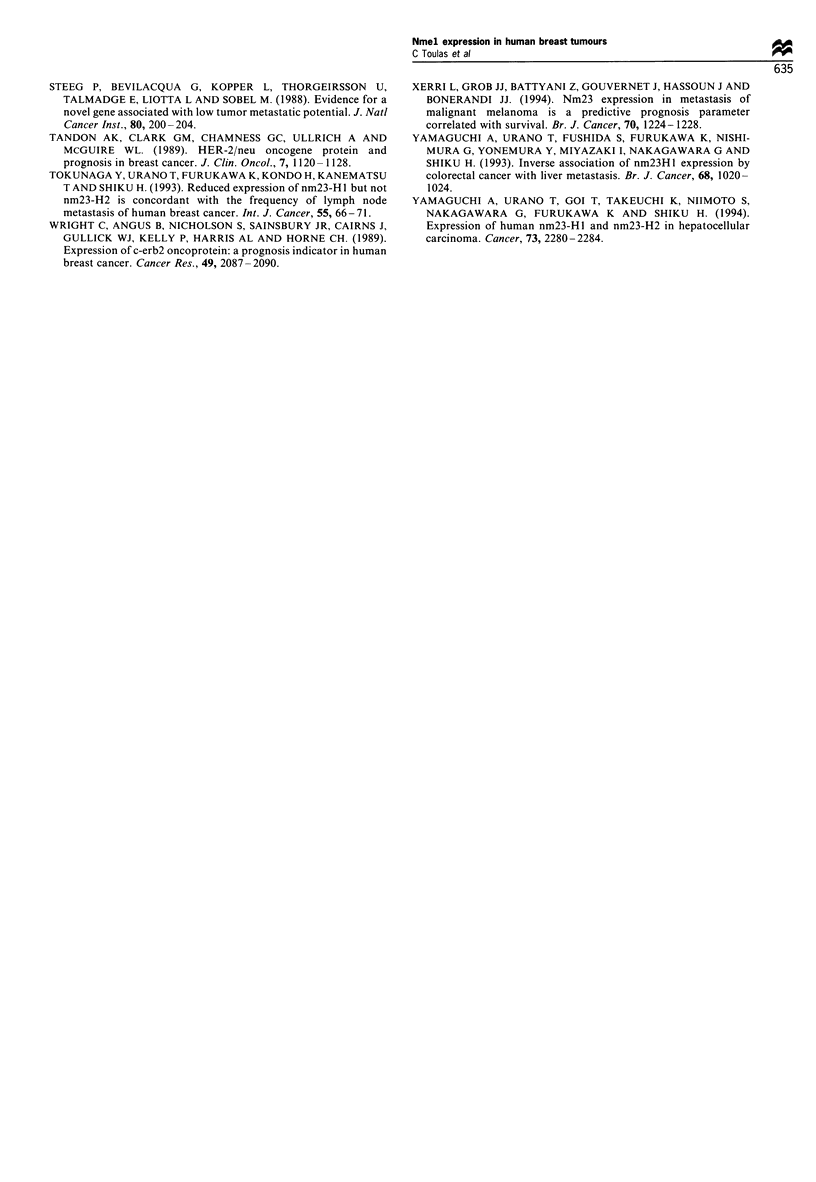# Potential prognostic value in human breast cancer of cytosolic Nme1 protein detection using an original hen specific antibody.

**DOI:** 10.1038/bjc.1996.109

**Published:** 1996-03

**Authors:** C. Toulas, J. Mihura, C. de Balincourt, B. Marques, E. Marek, G. Soula, H. Roche, G. Favre

**Affiliations:** Claudius Regaud Comprehensive Center, Toulouse, France.

## Abstract

**Images:**


British Journal of Cancer (1996) 73, 630-635
© 1996 Stockton Press All rights reserved 0007-0920/96 $12.00

### Potential prognostic value in human breast cancer of cytosolic Nmel protein detection using an original hen specific antibody

C Toulas[1], J Mihura[1], C de Balincourt[1], B Marques[1], E Marek[1], G Soula[1,2], H Roche[1] and G Favre[1,2]

[1]*Claudius Regaud Comprehensive Center, 20-24 rue du Pont St Pierre, 31052 Toulouse Cedex, France;* [2]*Laboratory of Drug Targeting Research, JE 175 DRED, Paul Sabatier University, Toulouse, France*

**Summary** The metastasis-suppressor *nme* gene (also called *nm23*), first identified in murine melanoma cells, exists as two forms in human: *nmel* and *nme2*. However, only the lack of expression of *nmel* has been related to distant metastasis appearance in human breast cancer. The aim of this work was first to raise specific antibodies to allow the analysis of Nmel and then, with this specific tool, to evaluate the predictive value of Nmel detection in cytosolic extracts of human breast tumours. We obtained a hen antibody that specifically reacts with Nmel without any cross-reaction with Nme2. We analysed the expression of the protein in 59 human breast tumours and found a significant relationship between this expression and oestrogen receptor status ($P<0.001$). Moreover, Nmel expression is related to metastasis-free survival ($P<0.001$) and survival of patients ($P<0.001$). The determination of Nmel expression in primary tumours using our antibody should be an interesting predictive test of the metastasis for clinical investigations.

**Keywords:** metastasis-suppressor gene *nmel*; Nmel specific antibody; Western blotting analysis; human breast tumour

The metastasis-suppressor gene, *nmel* was identified in 1988 (Steeg *et al.*, 1988) by differential hybridisation between low and high metastatic murine melanoma cell lines: the mRNA expression of this gene was found to be higher in the low metastatic cell line. In human, the *nme* gene family consists of two closely related genes: *nmel* and *nme2*, which respectively code for two different subunits (A and B) of nucleotide diphosphate kinases (NDP kinases) (Gilles *et al.*, 1991). These two subunits, which show more than 88% homology, associate together in different ratios according to tissue location, to form hexameric NDP kinases. This class of enzymes is involved in microtubule association (Nickerson and Wells, 1991) and G-protein regulation (Kimura and Shimada, 1988) but the precise molecular mechanism underlying the role of *nme* genes in metastasis dissemination is still unclear. The metastasis-suppressor activity of *nmel* seems to be correlated more with serine phosphorylation of Nmel protein (MacDonald *et al.*, 1993) than with kinase activity (Sastre-Garau *et al.*, 1992). A recent report (Howlett *et al.*, 1994) suggests that *nmel* may play an important role in the differentiation of human breast cancer. Moreover, Postel *et al.* (1993) has suggested that the Nme2 protein is a transcription factor for the c-*myc* oncogene.

The predictive value of *nme* detection has been evaluated by several authors in human tumours. Reduced expression of *nmel* has been found in ovarian carcinomas (Mandai *et al.*, 1994), in hepatocellular carcinoma (Nakayama *et al.*, 1992; Yamaguchi *et al.*, 1994; Boix *et al.*, 1994), in metastatic lymph nodes from patients with papillary carcinoma of the thyroid (Arai *et al.*, 1993) and in metastasis of malignant melanoma (Xerri *et al.*, 1994). Conflicting results have been obtained in colorectal tumorigenesis: Yamaguchi (1993) obtained lower amounts of *nmel* mRNA and protein in colorectal tumours associated with liver metastasis than in those without such metastasis while Myeroff *et al.* (1993) showed an increase of the mRNA level in both high- and low-metastatic potential tumours, suggesting that the activity of the *nme* gene could be tissue specific. In breast cancer a low level of *nmel* mRNA has been correlated with a high metastatic potential (Henessy *et al.*, 1991; Bevilacqua *et al.*, 1989) to a greater extent than *nme2* (Stahl *et al.*, 1991).

The *nmel* gene is frequently affected by loss of heterozygosity (LOH) (Leone *et al.*, 1991) but *nmel* LOH was not uniformly associated with low protein expression and poor prognosis (Cropp *et al.*, 1994). Studies using antibodies directed against NDP kinase A have been performed to measure Nmel protein expression in tumours but, probably because of the high sequence homology between NDP kinase A and B, and the poor predictive value of the B form, these studies produced controversial results (Sawan *et al.*, 1994; Lacombe *et al.*, 1991). The first aim of the current work was to raise an antibody that would specifically recognise Nmel protein without any cross-reaction with Nme2, to allow specific analysis of Nmel expression. With this tool, we could then evaluate the predictive value of Nmel expression: for this, we studied the relationship between Nmel expression and other prognostic factors such as menopausal status, tumour size, histological grade, lymph node status, hormone receptors, cathepsin D expression, pS2, c-*erb*B-2. We then related Nmel expression to disease-free survival and overall survival in patients.

### Materials and methods

### *Normal and tumour tissues*

Breast biopsy specimens were obtained from 59 patients with operable primary breast cancer who entered the Claudius Regaud Center between August 1989 and November 1990. The samples, collected after surgical removal, were stored in liquid nitrogen until use. Four specimens of normal breast tissue obtained from patients following mammary reduction were similarly treated.

The patients entering this study met the following criteria: primary breast cancer, availability of complete clinical, histological and biological information, no other primary cancer and no preliminary treatment. Patients underwent physical examination every 3 months for the first 2 years and every 6 months thereafter. Post-surgical treatment was planned according to prognostic factors and included chemotherapy, hormonetherapy and/or radiotherapy.

Correspondence: C Toulas
Received 22 March 1995; revised 21 September 1995 ; accepted 13 October 1995

*Pathology*

Tumour size was recorded as the largest diameter of the tumour. Modified Scarff, Bloom and Richardson grades (Contesso *et al.*, 1987) were assigned to all tumours by scoring tubular formation, nuclear pleomorphism and mitosis. Lymph node status was sought and histological examination used to confirm the involvement with tumour cells. The average number of lymph nodes examined was 15 and 30% of the patients showed no lymph node invasion.

*Synthesis of peptide antigen*

The peptide sequence of the antigen was designed from the amino acid sequence of NDP kinase A (Stahl *et al.*, 1991) to be divergent with the corresponding sequence of NDP kinase B: the 16 amino acid peptide from glycine 37 to tyrosine 53 of the protein was synthesised by Novabiochem (France), purified by high-performance liquid chromatography (HPLC) and coupled to haemocyanine to increase its immunoreactivity.

*Immunisation of the hen and antibody extraction*

The hen received a first injection in the peritoneal muscle of 10 $\mu$g of haemocyanin–peptide complex dissolved in 500 $\mu$l of complete Freund's adjuvant (Calbiochem, France). Two identical injections were then given 12 and 20 days later (Gassmann *et al.*, 1990). The eggs were collected daily and stored at 4°C until processing. Egg yolk was separated from the white, carefully washed with deionised water to remove albumin, diluted in 60 ml of a 3.5% polyethylene glycol solution (PEG 6000, Pharmacia, France), and centrifuged for 10 min at 14 000 ***g*** at 4°C. The supernatant was filtered and adjusted to 9% PEG 6000; the solution was centrifuged for 10 min at 9000 ***g*** at 4°C. The pellet was resuspended in 40 ml of 12% PEG and centrifuged for 10 min at 9000 ***g*** at 4°C. The final pellet was resuspended in 5 ml of phosphate-buffered saline (PBS) and stored at −20°C until use. In this solution, the IgY represented more than 80% of the total protein extracted and the specific antibody against NDP kinase A was 50% of the total IgY (Gassmann *et al.*, 1990). Protein concentration was measured according to the Bradford technique (Bradford, 1976).

*Analysis of antibody specificity*

The labelling specificity of the egg yolk antibody was analysed by Western blot. NDP kinase A and B, purified as described previously (Gilles *et al.*, 1991), were kindly provided by Dr Lascu (University of Bordeaux 2, France).

*Extraction of cytosols and membranes from tumours*

The breast tumour or normal breast tissue was homogenised in 10 mM Tris buffer pH 7.4 containing 20 mM molybdic acid, 12 mM monothioglycerol and centrifuged at 105 000 ***g*** for 30 min at 4°C. The supernatant represented the cytosolic extract. The pellet was resuspended in 5 mM Hepes, 3 mM EDTA, 1% Triton X100, 4 mM benzamidine, 1 mM DTT pH 7.4, and centrifuged for 30 min at 105 000 ***g***. This supernatant was the membrane extract. Protein concentrations were determined using the Bradford technique (Bradford, 1976) for cytosolic extract and bicinchoninic acid (Smith *et al.*, 1985) for the membrane extract.

*Measurement of oestrogen or progesterone receptors, cathepsin D and pS2*

The amount of hormonal receptors in the cytosolic extracts was determined using oestrogen receptor and progesterone receptor EIA kits (Abbott, France). The cathepsin D and pS2 cytosolic protein amounts were quantified using specific kits (CIS France).

*Measurement of the amount of erbB-2 protein*

Total protein (50 $\mu$g) from membrane extracts were diluted in 10 mM Tris HCl, 150 mM sodium chloride, 0.2% Tween20 pH 8 (TBST) and spotted onto nitrocellulose membrane (0.2 $\mu$m Biorad) under vacuum (Dot Blot system, Biorad, France). The membrane was then incubated at room temperature in TBST containing 1% lyophilised milk powder for 90 min and then for 1 h with the same solution containing the monoclonal antibody c-neu Ab3 (Oncogene Science, France) at 10 $\mu$g ml$^{-1}$. After three successive washes in TBST the nitrocellulose membrane was incubated in TBST containing 0.75 $\mu$g ml$^{-1}$ of peroxidase-conjugated goat anti-mouse antibody (Tebu, France) and washed again three times with TBST. After incubation in the ECL system (Amersham, France), the membrane was exposed for 5 s to Hyperfilm MP (Amersham, France). The film was scanned using a densitometer (Performance, Sebia, France). Variable amounts (from 10 $\mu$g to 60 $\mu$g) of protein extracted from MCF 7 membranes under the same conditions as the tumours, were loaded onto the same membrane. The signal was quantified using the densitometer and the calibration curve was then drawn. The amount of ErbB-2 protein contained in tumours was determined from this curve and expressed in arbitrary units determined as equivalent $\mu$g MCF 7 membrane protein per $\mu$g of tumour protein loaded.

*Western blot analysis of Nmel*

Total cytosolic protein (60 $\mu$g) from each sample and 1 $\mu$g of pure NDP kinase (Sigma, France) were loaded on a 15% polyacrylamide gel and electrophoresed at 150 V as described by Laemmli (1970). The gel was equilibrated in transfer buffer (480 mM Tris pH 8, 390 mM glycine, 0.5% sodium dodecyl sulphate (SDS), 25% methanol) for 10 min at room temperature. The transfer was then performed on a 0.2$\mu$m nitrocellulose membrane at 250 mA for 2 h in the transfer buffer. The membrane was incubated in TBST containing 1% lyophilised milk powder for 90 min, then in the same solution containing extracted anti-Nmel IgY (20 $\mu$g protein ml$^{-1}$) for 1 h. After three successive washes in TBST the membrane was incubated in TBST containing 1% milk and rabbit anti-chicken IgY peroxydase conjugates (Sigma, France) at a final dilution of 1:4000 for 1 h. Three washes were performed in TBST and the antigen was detected using the ECl system as previously described. The limit of detection was 200 ng of pure NDP kinase A (data not shown). The autoradiographs were scanned and the results expressed as positive if the scan could detect a band corresponding to a molecular weight of 18 kDa, and negative in the other cases. The pure NDP kinase (5 $\mu$g) loaded on the gel was used as an internal standard to avoid variations due to different exposures from one gel to another.

*Statistical analysis*

The association of Nmel expression with other discrete clinicobiological variables was assessed by chi-square analysis. The relationships between Nmel expression and cathepsin D, pS2 and c-erbB-2 were analysed as continuous variables using the Student's *t*-test. Disease-related death for specific survival and metastasis for metastasis-free survival were scored as events with censoring of other patients at the time of last follow-up. Survival curves were calculated by the method of Kaplan and Meier. Univariate analyses were performed with the log-rank test.

### Results

*Antibody specificity*

The aim of this work was to obtain an antibody able to react specifically with Nmel without any cross-reaction with the Nme2 form. Because of the high homology of the amino acid sequence (more than 88%) that exists between these two proteins, we decided to immunise the animals with a synthetic peptide whose sequence is localised in a region of weak homology. This 16 amino acid peptide of Nmel is located from glycine 37 to tyrosine 53 and represents less than 50% of sequence homology with the same region of Nme2.

We first tested the specificity of this antibody by Western blot analysis. As shown in Figure 1, Nmel antibody was able to detect pure NDP kinase A whereas it did not react with pure NDP kinase B. Control IgY did not reveal any signal (data not shown).

*Expression of Nmel in normal and tumour tissues*

The expression of Nmel in cytosolic extracts from normal breast tissues and tumours was then analysed. The Nmel antibody revealed a band corresponding to the molecular weight of NDP kinase A (18 kDa) (Figure 2) in breast tumours. This band was not present when the antibody was first incubated with 10 $\mu$g of peptide coupled to haemocyanin (Figure 2). No 18 kDa form of Nmel could be detected in normal breast tissue (data not shown). We then analysed the expression of Nmel in 59 human breast tumours by scanning the 18 kDa band on the Western blot. Our results are presented as 'Nmel + ' if the scan could detect a 18 kDa band and 'Nmel − ' in the other cases.

*Relationship between Nmel expression and other prognostic factors*

We analysed the relationships that might exist between Nmel expression and the other prognostic factors listed in Table I. No significant difference was detected between the expression of Nmel and menopausal status, nodal invasion or tumour size (Table I). Concerning the histological grade, 74% of well-differentiated tumours (grades 1 and 2) expressed Nmel but the relationship was not significant ($P$=7.2%) (Table I). A significant difference was noted according to oestrogen receptor status (ER): around 71% of ER-negative tumours did not express Nmel whereas 81% of ER-positive tumours expressed Nmel ($P$<0.001). Progesterone receptor (PR)-positive tumours preferentially expressed Nmel (74%) compared with PR-negative ones (50%) but the relationship was not significant (Table I). A possible relationship between Nmel and tumour hormone dependence was tested by analysing the pS2 protein expression level in Nmel+ tumours *vs* Nmel− (Table II). No significant difference could be observed in the pS2 protein level between Nmel+ tumours and Nmel− tumours. Similar results were obtained when comparing Nmel expression with the amount of cathepsin D and with erbB-2 expression: 17% of tumours showed an overexpression of the ErbB-2 protein level (higher than 30 units). However, no significant difference in ErbB-2 expression level was observed between Nmel− and Nmel+ tumours (Table II).

**Figure 1** Analysis of the hen antibody specificity: the Western blot analysis was performed on 4 $\mu$g of pure NDP Kinase B (lane 1) and 4 $\mu$g of pure NDP Kinase A (lane 2) as described in Materials and methods.

**Figure 2** Nmel expression in tumour tissues. The analysis was performed as described in Materials and methods, using 60 $\mu$g of cytosol from tumour tissues expressing Nme1 (lane 1) or not (lane 2) and pure Nme1 protein (lane 3). Preincubation of the antibody with 10 $\mu$g of antigen before Western blot analysis is shown in lane 4.

*Relationship between Nmel status, disease-free survival and overall survival*

Of the 59 patients entering this study, 14 have developed metastasis during the 50 months following surgery, and 12 died from cancer. Median follow-up was 42 months. Univariate analysis did not show any relationship between metastasis-free survival or specific survival and histological grade, lymph node invasion, hormone receptors, cathepsin D or ErbB-2. It confirmed the predictive value of tumour size ($P$<0.01) for specific survival and revealed that Nmel expression was significantly associated with longer metastasis-free survival ($P$<0.001) (Figure 3) and to overall survival ($P$<0.001) (Figure 4).

### Discussion

The results published by Steeg (1988) or Henessy (1991) suggested that *nmel* gene expression measured by mRNA analysis is related to the metastasis potential of human breast cancer. To analyse the potentialities of the tumour metastasis

**Table I** Relationship between Nmel expression and prognostic factors as discrete variables

| *Tumour and patient characteristics* | *Nmel −* | *Nmel +* | P |
|---|---|---|---|
| Nodal status | | | |
| Negative | 7 | 13 | NS |
| Positive | 13 | 26 | |
| Tumour grade | | | |
| 1 or 2 | 9 | 27 | NS |
| 3 | 11 | 12 | |
| Tumour size | | | |
| <2 cm | 4 | 13 | NS |
| >2 cm | 15 | 25 | |
| Menopausal status | | | |
| Premenopausal | 3 | 18 | NS |
| Post-menopausal | 9 | 23 | |
| Oestrogen receptor status | | | |
| Negative | 12 | 5 | <0.001 |
| Positive | 8 | 34 | |
| Progesterone receptor status | | | |
| Negative | 10 | 10 | NS |
| Positive | 10 | 29 | |

Statistical analysis was performed as described in Materials and methods. NS, non-significant relationship.

**Table II** Relationship between Nmel expression and prognostic factors as continuous variable

| *Tumour marker* | *Nmel−* | *Nmel+* | P |
|---|---|---|---|
| Cathepsin D level | | | |
| Mean | 48.2 | 42.2 | |
| s.d. | 36.6 | 22.7 | NS |
| *n* | 20 | 38 | |
| pS2 level | | | |
| Mean | 11.7 | 15.0 | |
| s.d. | 17.2 | 18.7 | NS |
| *n* | 20 | 38 | |
| ErbB-2 level | | | |
| Mean | 17.9 | 19.4 | |
| s.d. | 23.3 | 26.9 | NS |
| *n* | 15 | 27 | |

Statistical analysis was performed as described in Materials and methods. NS, non-significant relationship.

**Figure 3** Metastasis-free survival curve according to Nmel status: Nmel –, no band detectable at 18 kDa; Nmel +, a band detected by scan densitometer. The curve was calculated according to the Kaplan–Meier method and statistical analysis was performed as described in Materials and methods.

**Figure 4** Specific survival curves according to Nmel status: Nmel –, no band detectable at 18 kDa; Nmel +, a band detected by scan densitometer. The curve was calculated according to the Kaplan–Meier method and statistical analysis was performed as described in Materials and methods.

gene to predict metastasis outcome we raised an antibody against an Nmel protein. Instead of immunising animals with the whole Nmel protein a 16 amino acid peptide, derived from a region of the protein that represents a weak sequence homology, was injected into hens. The immunisation produced an antibody that specifically reacted with Nmel without any cross-reaction with the Nme2 form. This highly specific tool was then used to analyse Nmel protein expression in human breast tumours by Western blotting.

In our study, Nmel was not detected in any of the four normal breast tissues tested. Immunohistochemical studies of Nmel have given conflicting data concerning the staining of normal cells: in the study by Hiriyama *et al.* (1991) cells of normal acini and glands in the breast tissue generally could be weakly stained or not stained at all; Sastre-Gareau (1992), using a polyclonal anti-NDP kinase A, observed no or moderate staining of normal lobules and ducts; Simpson *et al.* (1994) reported that the percentage of Nm23 reactivity of lobular or ductal carcinoma *in situ* was greater than that of adjacent normal tissue. Other workers (Barnes *et al.*, 1991; Tokunaga *et al.*, 1993; Royds *et al.*, 1993), using different polyclonal antibodies, detected staining in normal breast. All the antibodies used in these studies recognised both Nmel and Nme2 and so detected the expression of both proteins in normal breast. That our antibody does not cross-react with Nme2 would explain the lower expression seen in our study; the level of Nmel expression in normal breast cytosol is probably lower than the sensitivity limit of our hen antibody.

Our work analysed the possible relationships between Nmel expression and other prognostic factors. No significant relationship was demonstrated either with age, menopausal status, tumour size, or lymph node status. Even if 74% of well-differentiated tumours expressed Nmel, no significant relationship could be detected between histological grade and Nmel expression ($P=7\%$). Studies by Henessy *et al.* (1991), Bevilacqua *et al.* (1989) and Royds *et al.* (1993) related Nmel negative expression to histological grade and to worsening invasive ductal carcinoma grade. It could be supposed that by using a larger population than examined in our study, we would have revealed a link between Scarff–Bloom-Richardson (SBR) grade and Nmel expression. On the other hand, a positive relationship between Nmel expression and hormonal receptors has been shown in our study but this relationship was only significant for the oestrogen receptor ($P<0.001$). Several workers (Adami *et al.*, 1985; Reiner, 1990) have related the amount of oestrogen receptor to the differentiation status of the tumour. Our data also suggest that *nmel* could be related to differentiation state of the tumour. This is in agreement with the recent observations of Howlett *et al.* (1994), who showed that *nmel*-transfected cell lines in culture with a reconstituted basement membrane presented characteristics of breast differentiation. Even if these results do not imply that the *nmel* gene universally controls breast differentiation, it may be one important gene in the process of differentiation.

Like the progesterone receptor, the expression of pS2 protein is oestrogen dependent (Brown *et al.*, 1984) and is predictive of a favourable response to endocrine therapy in human breast cancer (Henry *et al.*, 1991). In our work, no significant relationship was observed between Nmel expression and the pS2 protein level. These data and the absence of a link between Nmel expression and progesterone status would indicate that Nmel is not related to hormone dependent tumour status.

The c-*erb*B-2 oncogene, which encodes for a transmembrane glycoprotein with high homology with epidermal growth factor receptor, is amplified in 15–30% of breast cancer. This overexpression has been found to be related to an increase in the proliferative activity of the tumour and by some authors to a shorter survival (Wright *et al.*, 1989; Tandon *et al.*, 1989; Rilke *et al.*, 1991). More recently, an overexpression of c-erbB-2 has been associated to the response to chemotherapy (Muss *et al.*, 1994). A significant relationship between the expression of c-erbB-2 and the mRNA level of *nmel* has been shown in ovarian carcinomas (Mandai *et al.*, 1994); this relationship was also observed by immunohistochemical staining of sections for *nme* products and c-*erb*B-2. On the contrary, Slamon (1991) reported that human breast and ovarian carcinoma cell lines transfected with HER2/neu exhibited reduced *nme* mRNA levels. In our population, an overexpression of c-erbB-2 was observed in 17% of tumours but no significant relationship between c-erbB-2 and Nmel expression could be demonstrated. Furthermore, Nmel expression was independent of another metastasis predictive factor, cathepsin D levels, suggesting that the metastasis process involving Nmel protein could be different from that of cathepsin D.

We have described here highly significant relationships between Nmel expression and metastasis-free survival ($P<0.001$) and specific survival ($P<0.001$). These results obtained at the level of protein expression using our antibody are in agreement with several studies analysing *nmel* mRNA levels in breast cancer (Henessy *et al.*, 1991) and in other locations (Arai *et al.*, 1993). Although this approach needs to be confirmed by a larger study, it underlines the interesting role of specifically detecting cytosolic *nmel* for the prediction of metastasis outcome in breast cancer.

**Acknowledgements**
We thank Dr Lascu (University of Bordeaux II, France) and Dr Lidereau (Centre René Huguenin, St Cloud, France) for reading the manuscript, Dr Vachaud for his efficient cooperation in normal tissue collection and F Cheutin for her technical assistance. This work was supported by the Fédération Nationale des Centres de Lutte Contre le Cancer (Comité Midi-Pyrénées).

### References

ADAMI HO, GRAFFMAN S, LINDGREN A AND SALLSTROEM J. (1985). Prognostic implication of estrogen receptor content in breast cancer. *Breast Cancer Res. Treat.*, **5**, 293–297.

ARAI T, WATANABE M, ONODERA M, YAMASHITA T, MASUNAGA T, ITOYAMA S, ITOH K AND SUGAWARA I. (1993). Reduced nm23-H1 messenger RNA expression in metastatic lymph node from patients with papillary carcinoma of the thyroid. *Am. J. Pathol.*, **142**, 1938–1944.

BARNES R, MASOOD S, BARKER E, ROSENGARD AM, COGGIN DL, CROWELL T, KING CR, PORTER-JORDAN K, WARGOTZ ES, LIOTTA LS AND STEEG PS. (1991). Low nm23 expression in infiltrating ductal breast carcinomas correlates with patient survival. *Am. J. Pathol.*, **139**, 245–250.

BEVILACQUA G, SOBEL ME, LIOTTA LA AND STEEG PS. (1989). Association of low nm23 RNA levels in human primary infiltrating ductal breast carcinomas with lymph node involvement and other histopathologic indicators of high metastatic potential. *Cancer Res.*, **49**, 5185–5190.

BOIX L, BRUIX J, CAMPO J, SOLE M, CASTELLS A, FUSTER J, RIVERA F, CARDESA A AND RODES J. (1994). Nm23-H1 expression and disease recurrence after surgical resection of small hepatocellular carcinoma. *Gastroenterology*, **107**, 486–491.

BRADFORD MM. (1976). A rapid and sensitive method for the quantitation of microgram of protein utilizing the principle of protein dye binding. *Anal. Biochem.*, **72**, 248–254.

BROWN AM, JELSTCH JM, ROBERTS M AND CHAMBON P. (1984). Activation of pS2 gene transcription in primary response to estrogen in a human breast cancer cell line MCF7. *Proc. Natl Acad. Sci. USA*, **81**, 6344–6348.

CONTESSO G, MOURIESSE H, FRIEDMAN S, GENIN J, SARRAZIN D AND ROUESSE J. (1987). The importance of histologic grade in long term prognosis of breast cancer. A study of 1010 patients uniformly treated at the Institut Gustave Roussy. *J. Clin. Oncol.*, **5**, 1378–1386.

CROPP C, LIDEREAU R, LEONE A, LISCIA D, CAPPA A, CAMPBELL G, BARKER E, LEDOUSSAL V, STEEG P AND CALLAHAN R. (1994). Nme1 protein expression and loss of heterozygosity mutations in primary human breast tumors. *J. Natl Cancer Inst.*, **86**, 1167–1169.

GASSMANN M, THOMMES P, WEISER T AND HUBSCHER U. (1990). Efficient production of chicken egg yolk antibodies against a conserved mammalian protein. *FASEB J.*, **4**, 2528–2532.

GILLES AM, PRESECAN E, VONICA A AND LASCU I. (1991). Nucleoside diphosphate kinase from human erythrocytes. Structural characterization of the two polypeptide chains responsible for heterogeneity of the hexameric enzyme. *J. Biol. Chem.*, **266**, 8784–8789.

HENESSY C, HENRY JA, MAY F, WESTLEY B, ANGUS B AND LENNARD T. (1991). Expression of the metastatic gene nm23 in human breast cancer: an association with good prognosis. *J. Natl Cancer Inst.*, **83**, 281–285.

HENRY JA, PIGGOTT NH, MALLICK UK, NICHOLSON S, FARNDON JR, WESTLEY BR AND MAY FEB. (1991). pNR-2/pS2 immunohistochemical staining in breast cancer: correlation with prognostic factors and endocrine response. *Br. J. Cancer*, **63**, 615–622.

HIRAYAMA R, SAWAI S, TAKAGI Y, MISHIMA Y, KIMURA N, SHIMADA N, ESAKI Y, KURASHIMA C, UTSUYAMA M AND HIROKAWA K. (1991). Positive relationship between expression of anti-metastatic factor (nm23 gene product or nucleoside diphosphate kinase) and good prognosis in human breast cancer. *J. Natl Cancer Inst.*, **83**, 1249–1250.

HOWLETT A, PETERSEN O, STEEG P AND BISELL M. (1994). A novel function for the nm23-H1 gene: overexpression in human breast carcinoma cells leads to the formation of basement membrane and growth arrest. *J. Natl Cancer Inst.*, **86**, 1838–1844.

KIMURA N AND SHIMADA N. (1988). Membrane-associated nucleoside diphosphate kinase from rat liver. *J. Biol. Chem.*, **263**, 4647–4653.

LACOMBE M-L, SASTRE-GARAU X, LASCU I, VONICA A, WALLET V, THIERY JP AND VERON M. (1991). Overexpression of nucleoside diphosphate kinase (nm23) in solid tumors. *Eur. J. Cancer*, **27**, 1302–1307.

LAEMMLI UK. (1970). Cleavage of structural proteins during the assembly of the head of bacteriophage T4. *Nature*, **27**, 680–685.

LEONE A, WESLEY MCBRIDE O, WESTON A, WANG M, ANGLARD P, CROPP C, GOEPEL J, LIDEREAU R, CALLAHAN R, MARSTON LINEHAN W, REES RC, HARRIS CC, LIOTTA L AND STEEG P. (1991). Somatic allelic deletion of nm23 in human cancer. *Cancer Res.*, **51**, 2490–2493.

MACDONALD NJ, DE LA ROSA A, BENEDICT MA, FREIDJE JM, KRUTSCH H AND STEEG P. (1993). A serine phosphorylation of Nm23 and not its nucleoside diphosphate kinase activity, correlates with suppression of tumor metastasis potential. *J. Biol. Chem.*, **268**, 25780–25789.

MANDAI M, KONISHI I, KOSHIYAMA M, MORI T, ARAO S, TASHIRO H, OKAMURA H, NOMURA H, HIAI H AND FUKUMOTO M. (1994). Expression of metastasis related nm23H1 and nm23H2 genes in ovarian carcinomas: correlation with clinicopathology, EGFR, c-erbB2, and c-erbB3 genes and sex steroid receptor expression. *Cancer Res.*, **54**, 1825–1830.

MUSS HB, THOR AD, BERRY DA, KUTE T, LIU ED, KOERNER F, CIRRINCIONE CT, BUDMAN DR, WOOD WC, BARCOS M AND HENDERSON IC. (1994). c-erbB2 expression and response to adjuvant therapy in women with node-positive early breast cancer. *N. Engl. J. Med.*, **330**, 1260–1266.

MYEROFF LL AND MARKOWITZ SD. (1993). Increased nm23 H1 and nm23H2 messenger RNA expression and absence of mutations in colon carcinomas of low and high metastatic potential. *J. Natl Cancer Inst.*, **85**, 147–152.

NAKAYAMA T, OHTSURA A, NAKAO K, SHIMA M, NAKATA K, WATANABE K, ISHII N, KIMURA N AND NAGATAKI S. (1992). Expression in human hepatocellular carcinoma of nucleoside diphosphate kinase, a homologue of the nm23 gene product. *J. Natl Cancer Inst.*, **84**, 1349–1354.

NICKERSON JA AND WELLS WW. (1984). The microtubules-associated nucleoside diphosphate kinase. *J. Biol. Chem.*, **259**, 11297–11304.

POSTEL EH, BERBERICH SJ, FLINT SJ AND FERRONE CA. (1993). Human c-myc transcription factor Puf identified as nme2 nucleoside diphosphate kinase, a candidate suppressor of tumor metastasis. *Science*, **261**, 428–429.

REINER A. (1990). Histopathologic characterization of human breast cancer in correlation with estrogen receptor status. *Cancer* **61**, 1149–1154.

RILKE F, COLNAGHI MI, CASCINELLI N, ANDREOLA S, BALDINI MT, BUFALINO R, DELLA PORTA G, MENARD S, PIEROTTI MA AND TESTORS A. (1991). Prognosis significance of HER/Neu expression in breast cancer and its relationship to other prognosis factors. *Int. J. Cancer*, **49**, 44–49.

ROYDS JA, STEPHENSON TJ, REES RC, SHORTHOUSE AJ AND SILCOCKS PB. (1993). Nm23 protein expression in ductal in situ and invasive breast carcinoma. *J. Natl Cancer Inst.*, **85**, 727–731.

SASTRE-GARAU X, LACOMBE ML, JOUVE M, VERON M AND MAGDALENAT H. (1992). Nucleoside diphosphate kinase/NM23 expression in breast cancer: lack of correlation with lymph node metastasis. *Int. J. Cancer*, **50**, 533–538.

SAWAN A, LASCU I, VERON M, ANDERSON JJ, WRIGHT C, HORNE C AND ANGUS B. (1994). NDP-K/nm23 expression in human breast cancer in relation to relapse survival, and other prognostic factors: an immunohistochemical study. *J. Pathol.*, **172**, 27–34.

SIMPSON JF, O'MALLEY F, DUPONT WD AND PAGE DL. (1994). Heterogeneous expression of nm23 gene product in noninvasive breast carcinoma. *Cancer*, **73**, 2352–2358.

SLAMON D. (1991). Expression of the nm23 gene and breast cancer prognosis. *J. Natl Cancer Inst.*, **83**, 229–231.

SMITH PK, KROHN RI, HERMANSON GT, MALLIA AK, GARTNER FH, PROVENZANO MD, FUJIMOTO EK, GOEKE NM, OLSON BJ AND KLENK DC. (1985). Measurement of protein using bicinchoninic acid. *Anal. Biochem.*, **150**, 76–85.

STAHL J, LEONE A, ROSENGARD A, PORTER L, KING R AND STEEG P. (1991). Identification of a second human nm23 gene, nm23-H2. *Cancer Res.*, **51**, 445–449.

STEEG P, BEVILACQUA G, KOPPER L, THORGEIRSSON U, TALMADGE E, LIOTTA L AND SOBEL M. (1988). Evidence for a novel gene associated with low tumor metastatic potential. *J. Natl Cancer Inst.*, **80,** 200–204.

TANDON AK, CLARK GM, CHAMNESS GC, ULLRICH A AND MCGUIRE WL. (1989). HER-2/neu oncogene protein and prognosis in breast cancer. *J. Clin. Oncol.*, **7,** 1120–1128.

TOKUNAGA Y, URANO T, FURUKAWA K, KONDO H, KANEMATSU T AND SHIKU H. (1993). Reduced expression of nm23-H1 but not nm23-H2 is concordant with the frequency of lymph node metastasis of human breast cancer. *Int. J. Cancer*, **55,** 66–71.

WRIGHT C, ANGUS B, NICHOLSON S, SAINSBURY JR, CAIRNS J, GULLICK WJ, KELLY P, HARRIS AL AND HORNE CH. (1989). Expression of c-erb2 oncoprotein: a prognosis indicator in human breast cancer. *Cancer Res.*, **49,** 2087–2090.

XERRI L, GROB JJ, BATTYANI Z, GOUVERNET J, HASSOUN J AND BONERANDI JJ. (1994). Nm23 expression in metastasis of malignant melanoma is a predictive prognosis parameter correlated with survival. *Br. J. Cancer*, **70,** 1224–1228.

YAMAGUCHI A, URANO T, FUSHIDA S, FURUKAWA K, NISHIMURA G, YONEMURA Y, MIYAZAKI I, NAKAGAWARA G AND SHIKU H. (1993). Inverse association of nm23H1 expression by colorectal cancer with liver metastasis. *Br. J. Cancer*, **68,** 1020–1024.

YAMAGUCHI A, URANO T, GOI T, TAKEUCHI K, NIIMOTO S, NAKAGAWARA G, FURUKAWA K AND SHIKU H. (1994). Expression of human nm23-H1 and nm23-H2 in hepatocellular carcinoma. *Cancer*, **73,** 2280–2284.